# Healthcare-seeking behavior among people with HIV undergoing TB screening during the COVID-19 pandemic

**DOI:** 10.5588/ijtldopen.24.0111

**Published:** 2024-07-01

**Authors:** I. Aggarwal, L.H. Chaisson, B. Opira, D.W. Dowdy, P.P.J. Phillips, F.C. Semitala, C. Yoon

**Affiliations:** ^1^ University of Illinois College of Medicine, Chicago, IL, USA;; ^2^ Center for Global Health, University of Illinois at Chicago, Chicago, IL, USA;; ^3^ Division of Infectious Diseases, Department of Medicine, University of Illinois at Chicago, Chicago, IL, USA;; ^4^ Infectious Diseases Research Collaboration, Kampala, Uganda;; ^5^ Department of Epidemiology, Johns Hopkins Bloomberg School of Public Health, Baltimore, MD, USA;; ^6^ Division of Pulmonary and Critical Care Medicine, University of California San Francisco, San Francisco, CA, USA;; ^7^ UCSF Center for Tuberculosis, University of California San Francisco, San Francisco, CA, USA;; ^8^ Department of Medicine, Makerere University College of Health Sciences, Kampala, Uganda;; ^9^ Makerere University Joint AIDS Program, Kampala Uganda

**Keywords:** tuberculosis, healthcare-seeking behavior, Uganda, stigma, barriers to care.

Dear Editor,

The COVID-19 pandemic had a damaging impact on the delivery of TB services, reversing years of progress and derailing global TB targets.^1,2^ Both the pandemic and the response to the pandemic led to disruptions in every step in the TB care cascade, with a particularly severe impact on the first step of the cascade: access to diagnostic services.^3,4^ As a result, 18% fewer people were diagnosed with TB worldwide in 2020 compared to 2019, with the greatest deficits in high-burden areas.^4,5^ In 2020, among people with HIV (PWH), TB detection and notification is estimated to have declined by close to 10% compared to 2019.^6^ In Uganda, a high TB-HIV burden country, following an initial 10-week nationwide lockdown (March 25–May 26, 2020), TB case notifications decreased by up to 22%.^7^ A second nationwide lockdown (June 7–July 19, 2021) caused a similar reduction in TB case notifications.^7^ Although TB case notifications worldwide have since rebounded to near pre-pandemic levels, delayed and missed TB diagnoses due to COVID-19 are thought to have caused global TB incidence to rise by 3.6% between 2020 and 2021, reversing declines of 1-2% per year seen over the previous two decades.^2,3^ To investigate the factors associated with this delayed entry into care and TB screening among PWH, we evaluated healthcare-seeking behavior among individuals newly entering HIV care in Kampala, Uganda, immediately following Uganda’s second nationwide lockdown.

Data for this analysis were from an ongoing phase 3 randomized controlled trial comparing two TB screening strategies (ClinicalTrials.gov NCT04557176). For the trial, trained study staff collected demographic and clinical data from outpatient adults with HIV initiating antiretroviral therapy (ART) at four HIV clinics.^8^ At enrollment, participants completed a standardized questionnaire about healthcare-seeking behavior, barriers to seeking care, and history of COVID-19 testing and vaccination. Here, we included trial participants enrolled from August 2021 to September 2022. We assessed participant characteristics, self-reported barriers to care, and the proportion of participants who delayed care for >1 month. We compared characteristics and TB status of those who delayed care for >1 month vs. those who sought care within 1 month using χ^2^ square and Wilcoxon rank-sum tests. This study was approved by the Institutional Review Boards of the University of California San Francisco, San Francisco, CA, USA; and Makerere University, Kampala, Uganda. All participants provided written informed consent.

From August 2021 to September 2022, we enrolled 1,097 PWH initiating ART, of whom 689 (62.8%) were female, median age was 30 years (interquartile range [IQR] 26–38), and median pre-ART CD4 cell count was 312 cells/µL (IQR 114–407) (Table). A total of 127 (11.6%) were diagnosed with TB disease at baseline. Overall, 195 (17.8%) participants reported having previously been tested for COVID-19, 16 (1.5%) reported a prior diagnosis of COVID-19, and 414 (37.7%) reported receiving at least one dose of a vaccine for COVID-19. A total of 812 (74.0%) reported experiencing barriers to seeking care. The most reported barriers to care included work obligations (*n* = 455, 41.5%), transportation costs (*n* = 88, 8.0%), and fear of being diagnosed with HIV or experiencing stigma or discrimination (*n* = 112, 10.2%). More than one-third (*n* = 374, 34.1%) of participants reported delaying care for >1 month. Demographics, clinical characteristics and TB prevalence were similar between participants who did vs. did not delay care for >1 month. Compared to those who sought care within 1 month, a higher proportion of those who delayed care for >1 month had previously been tested for COVID-19 (23.8% vs. 14.7%; *P* < 0.001), and higher proportions reported that COVID-19-related movement restrictions (5.6% vs. 0.6%; *P* < 0.001), transportation costs (11.8% vs. 6.1%; *P* < 0.001), and fear of HIV diagnosis or stigma (18.7% vs. 5.8%; *P* < 0.001) contributed to their delay in seeking care.

**Table.** Demographics, baseline characteristics, and healthcare-seeking behavior of adults new to HIV care undergoing TB screening, August 2021–September 2022.

Baseline characteristics	Total (*n* = 1,097)* *n* (%)	Delayed care >1 month (*n* = 374) *n* (%)	Delayed care <1 month (*n* = 721) *n* (%)	*P*-value^†^
Female sex	689 (62.8)	244 (65.2)	443 (61.4)	0.24
Age, years, median [IQR]	30 [26–38]	30 [25–32]	30 [25–37]	0.34
CD4 cell count, cells/μL, median [IQR]	312 [114–407]	246 [101–405]	242 [117–411]	0.68
BMI, m/kg^2^, median [IQR]	22.5 [20.2–25.3]	22.5 [20.2–25.5]	22.4 [20.2–25.3]	0.79
Prevalent TB disease^‡^	127 (11.6)	47 (12.6)	80 (11.1)	0.53
Educational attainment				0.005
None	73 (6.7)	36 (9.6)	37 (5.1)	
Primary	485 (44.2)	164 (43.9)	319 (44.2)	
Secondary	464 (42.3)	145 (38.8)	319 (44.2)	
High school	19 (1.7)	12 (3.2)	7 (1.0)	
University	26 (2.4)	6 (1.6)	20 (2.8)	
Graduate school	6 (0.5)	3 (0.8)	3 (0.4)	
Vocational school	24 (2.2)	8 (2.1)	16 (2.2)	
Household size, *n*, median [IQR]	3 [2–4]	3 [2–4]	3 [2–4]	0.32
Employed	797 (72.7)	268 (71.7)	527 (73.1)	0.66
Weekly household income, USD, median [IQR]	22 [13.5–43]	21.5 [12.9–40.3]	21.5 [13.4–45.7]	0.19
COVID-19 history				
Previously tested for COVID-19	195 (17.8)	89 (23.8)	106 (14.7)	<0.001
Previously diagnosed with COVID-19	16 (1.5)	5 (1.3)	11 (1.5)	1.00
Received ≥1 dose of COVID-19 vaccine	414 (37.7)	136 (36.4)	277 (38.4)	0.55
Barriers to seeking care				
COVID-19-related movement restrictions	25 (2.3)	21 (5.6)	4 (0.6)	<0.001
Fear of becoming infected with COVID-19	27 (2.5)	13 (3.5)	14 (1.9)	0.18
Work obligations	455 (41.5)	169 (45.2)	285 (39.5)	0.08
Child or elder care obligations	18 (1.6)	7 (1.9)	11 (1.5)	0.86
Transportation costs	88 (8.0)	44 (11.8)	44 (6.1)	<0.001
Healthcare costs	26 (2.4)	9 (2.4)	17 (2.4)	1.00
Fear of HIV diagnosis, stigma, or discrimination	112 (10.2)	70 (18.7)	42 (5.8)	<0.001
Previously sought care at a private clinic or pharmacy, or self-medicated	28 (2.6)	13 (3.5)	15 (2.1)	0.43
Other	75 (6.8)	28 (7.5)	47 (6.5)	0.63

* Two participants did not specify how long they waited to seek care.

† Calculated from χ^2^ tests and Wilcoxon rank-sum tests.

‡ Prevalent TB defined as urine lipoarabinomannan- or sputum Xpert^®^

MTB/RIF Ultra-positive TB.

IQR = interquartile range; BMI = body mass index; USD = US dollar.

In this analysis of PWH newly entering care in Uganda 15 months into the pandemic, TB prevalence was high (12%) and delays in seeking care were common, with 34% of participants delaying care for >1 month. Barriers to care that were not specific to COVID-19, including work obligations, transportation costs and HIV-related stigma, were frequently cited as reasons for delaying care. Although COVID-19 related barriers were less commonly reported in the overall study population, movement restrictions resulting from COVID-19 were associated with delaying care for >1 month. Pre-COVID-19 qualitative analyses consistently demonstrated that resource availability, costs and stigma were major drivers of delayed entry into care in high TB/HIV burden settings, and our findings suggest that these factors continue to affect healthcare-seeking among adults with HIV in Uganda.^9–12^ This demonstrates the importance of maintaining disease-specific and systems-oriented initiatives related to combatting stigma and barriers to access during times of acute public health crisis.

Importantly, between August 2021 and September 2022, our study revealed that only approximately 18% of participants had been tested for COVID-19 and 38% had been vaccinated for COVID-19. These low rates of testing and vaccine uptake are consistent with prior studies from sub-Saharan Africa,^10–12^ where access to testing and vaccination has lagged relative to middle- and high-income settings due to numerous structural, resource and implementation challenges,^10,12,13^ alongside fear of discrimination and stigma.^9–11,14^ Our results support the need for interventions to address these multifaceted reasons for delaying care to ensure timely access to HIV, TB, and COVID-19 care.

This study has several limitations. First, we included adults presenting for HIV care in Kampala, Uganda and do not have data on barriers for individuals who never reached care, or from settings outside of Uganda, which may limit generalizability. Data from national TB prevalence surveys suggest that many individuals eligible for TB screening delay or do not seek care; identifying and addressing barriers to care among these individuals at high TB risk remains a public health priority.^15^ Second, data on healthcare-seeking behavior were self-reported and may have been imprecise if participants could not recall or did not disclose how long they had delayed care and/or all barriers encountered. Third, we included individuals who presented to care from August 2021 through September 2022; barriers may have differed prior to or following this period, and may have changed during this period as the pandemic evolved. Given that this analysis focused on the period after the second major lockdown, it may underrepresent barriers to care that are specific to COVID-19 earlier in the pandemic. Despite these limitations, our analysis provides valuable data on barriers to TB-HIV care in Uganda during the COVID-19 pandemic.

In conclusion, although the COVID-19 pandemic created numerous new obstacles to TB-HIV care, traditional barriers remained common and may have been exacerbated. In times of acute public health crisis, it is imperative that initiatives targeting common barriers to care are maintained and strengthened. To promote timely TB and HIV diagnosis and treatment initiation, a continued focus on interventions that address socioeconomic barriers and stigma are needed in high TB-HIV burden settings.
